# Bioethical differences between drug addiction treatment professionals inside and outside the Russian Federation

**DOI:** 10.1186/1477-7517-8-15

**Published:** 2011-06-10

**Authors:** Vladimir D Mendelevich

**Affiliations:** 1 Kazan State Medical University, Department of Medical and General Psychology, 49 Butlerova Street, Kazan 420012, Russia

## Abstract

This article provides an overview of a sociological study of the views of 338 drug addiction treatment professionals. A comparison is drawn between the bioethical approaches of Russian and foreign experts from 18 countries. It is concluded that the bioethical priorities of Russian and foreign experts differ significantly. Differences involve attitudes toward confidentiality, informed consent, compulsory treatment, opioid agonist therapy, mandatory testing of students for psychoactive substances, the prevention of mental patients from having children, harm reduction programs (needle and syringe exchange), euthanasia, and abortion. It is proposed that the cardinal dissimilarity between models for providing drug treatment in the Russian Federation versus the majority of the countries of the world stems from differing bioethical attitudes among drug addiction treatment experts.

## Introduction

Russian and international narcology (addiction medicine) began to develop along divergent paths during the second half of the twentieth century. Indeed, Russian narcology has ceased to be a part of international narcology. There are cardinal differences in current scientific views on the nature of dependency, its neurobiological or psychopathological bases, and on standards of therapy and how best to organize narcological care [1-9]. In 1976 Soviet narcology cut "the umbilical cord" connecting it with psychiatry and began to be seen as an independent discipline. No longer was it considered necessary for experts to seek an underlying psychiatric cause to addiction. In contrast, in the vast majority of countries, narcology evolved within the boundaries of psychiatry. There is evidence to suggest that a principal cause of the current differences between Russian and international narcology is the distance placed between Soviet narcology and psychiatry, a breach that paved the way toward repressive measures against patients.

In parallel with these trends in Russian narcology there has been a gradual assessment of bioethical and deontological norms [6,10]. Although there have been calls for adherence to the principles of medical ethics, the procedures put into practice continue to be incompatible with these principles. Drug addicts still have even fewer patient rights than the mentally ill. They have been exposed to discrimination and stigmatization both in society and within drug treatment facilities. They have quite often been perceived by physicians as deviants or criminals, since during the Soviet period they were subject to measures of compulsion, isolation and re-education.

Contemporary Russian narcology is one of the few medical specialities in which doctors' and health care workers' ethical and deontological priorities still guide the processes of diagnosis, therapy and rehabilitation [11]. It is not unusual for an adolescent who has been caught taking drugs to be forced to register with a narcology clinic and deprived of rights. The choice of what therapeutic or rehabilitative approach to use (including those that involve compulsion) is often made on the false premise that drug addiction is not a disease, but a form of deviant behavior. In spite of the fact that narcology has a psychiatric component, the standards of bioethics and medical law that are observed in psychiatry [12-17] are not always applied to it. The legitimacy and expediency of applying the norms dictated by the Russian Federation law "Concerning Psychiatric Care and Guaranteeing Patient Rights" [18] to drug addicts are still being debated today [19,20].

This can probably be seen as stemming from the very different attitudes doctors have toward mental illness and the disease of drug addiction and, as a consequence, toward observing standard bioethical principles. According to sociological research [5], a large number (almost half) of Russian psychiatrists working in the area of narcology unequivocally believe that drug dependence and alcoholism are not mental disorders or diseases but are rather caused by "dissoluteness." Probably for this reason 54.5% of experts in drug addiction treatment identify religion as "the most effective method for treating drug addiction."

The hypothesis underlying the present study is that Russian and foreign drug treatment professionals configure their bioethical priorities very differently. In this connection, its method applied a sociological approach to studying the specific attitudes held by Russian and foreign specialists working in drug treatment toward various actual questions of contemporary bioethics and medical law. Some of the questions had to do with contentious issues of bioethics in general and some were specific to psychiatry and narcology. Among the former were questions relating to euthanasia, abortions, gender reassignment operations, cloning, the use of placental stem cells, organ transplantation, placebo-controlled clinical trials, the responsibility of HIV+ patients for their own illness, and Harm Reduction programs (the exchange of needles and syringes, recommendations about condom use). Among the latter were questions connected with respondent attitudes toward compulsory treatment in narcology, compulsory (obligatory) testing of students and schoolchildren for the use of psychoactive substances, opioid substitution therapy, the permissibility of disclosing confidential information or infringing the principle of informed consent in psychiatry and narcology, and the permissibility of preventing the mentally ill from having children.

To carry out the study, a questionnaire consisting of 19 questions both a Russian and English version (I would like to express my gratitude to IHRD for help translating the questionnaire) was created. Research was conducted anonymously. The respondents were professionals actively engaged in providing narcological care (psychiatrists, experts in drug addiction treatment) who wished to take part in the study. (Under the circumstances, it was not deemed necessary to obtain informed consent.) Respondents were informed that "Answers will be kept anonymous" and were encouraged to be "maximally candid" in their answers. The questionnaire was sent via e-mail, and answers were also received via e-mail. Questionnaires were sent to narcological clinics and centers in various regions of Russia, departments of psychiatry, narcology, and psychotherapy in the country's medical universities, and also to addiction, psychiatric, and medical associations, university departments of psychiatry, and addiction treatment centers located in various countries. In total, more than 1000 questionnaires were distributed (700 across the Russian Federation and nearly 300 internationally). A total of 264 completed Russian-language questionnaires and 92 English-language questionnaires were returned by respondents to researchers. Completed questionnaires were received from 18 countries: Australia, Belgium, Brazil, Great Britain, Vietnam, Germany, Israel, Italy, Canada, China, Latvia, Macedonia, the Netherlands, the United States, Thailand, France, Croatia, and Montenegro. All 246 Russian and 92 English questionnaires appeared to be correctly completed and were therefore submitted for statistical processing. The study sample consisted of 338 persons: 138 men (40.8%) and 200 women (59.2%). Tenure in the field ranged from 1 to 30 years. Results were accepted on a non-selective basis.

## Results and discussion

respondent attitudes toward the various controversial topics of bioethics and the medical law - were distributed as follows (Table 1).

**Table 1:** Respondent Attitudes toward Various Bioethical Problems

	Russian (n = 246)			Foreign (n = 92)		
	**Support**	**Do Not Support**	**Can't Say**	**Support**	**Do Not Support**	**Can't Say**

1. Do you support or not support the idea of **legalizing euthanasia**, when a terminally ill patient, after consultation with a committee of treating doctors, is allowed to decide to voluntarily end his life?	41.5%**	38.6%	19.9%	69.6%	13.0%	17.4%

2. Do you support or not support the procedure for **gender reassignment surgery for a person **who is diagnosed with transsexualism, and who has no psychological problems?	61.0%*	27.6%	11.4%	76.1%	13.0%	10.9%

3. Is **Homosexuality**, from your point of view, an illness that requires treatment, or not an illness?	21.1%	62.2%	16.7%	13.0%	76.1%	10.9%

4. What is your attitude to **experiments on human cloning**?	27.6%	44.3%	28.1%	32.6%	37.0%	30.4%

5. Do you think it's acceptable **to terminate a pregnancy (abort) **in the early stages, at the woman's request, when there are no medical reasons for terminating the pregnancy?	77.7%**	15.0%	7.3%	95.7%	4.3%	0%

6. In your opinion, is the procedure for extracting **placental stem cells **morally permissible or impermissible?	70.3%*	12.6%	17.1%	87.0%	4.3%	8.7%

7. In your opinion, should we ease access to use of **narcotic analgesics **for oncology patients suffering from pain?	87.0%*	8.5%	4.5%	97.8%	2.2%	0%

8. From your point of view, is an **HIV-positive patient **responsible or not responsible for his illness?	19.5%	43.1%	37.4%	10.9%	47.8%	41.3%

9. Do you think that the **transplant of organs **donated by a recently deceased person is morally permissible or not permissible?	86.2%	6.1%	7.7%	93.5%	4.3%	2.2%

10. In your opinion, do you think it is morally permissible to force those suffering from alcoholism or drug addiction to undergo **compulsory treatment **for their medical condition?	62.6% ***	29.3%	8.1%	28.3%	71.7%	0%

11. Do you support, or not support, the idea that **mentally ill women should be forbidden to have children **and should be forced to undergo sterilization?	40.6% ***	37.8%	21.6%	10.9%	78.3%	8.6%

12. What is your attitude toward the adoption of **substitution therapy for drug addiction**, which presupposes that the patients, for the management of their medical condition, will receive a prescription for medicines that contain narcotic substances?	51.2% ***	31.3%	17.5%	93.5%	4.3%	2.2%

13. Do you find it morally permissible or impermissible to pursue the prevention of the spread of sexually transmitted HIV infection among teenagers by **actively propagandizing "safe sex" **practices through use of condoms?	89.8%	4.5%	5.7%	93.5%	2.2%	4.3%

14. Do you find it morally permissible or impermissible to pursue the prevention of the spread of HIV infection through drug injection among drug addicts, by providing access to a **needle and syringe exchange**?	73.5%*	16.7%	9.8%	87.0%	8.7%	4.3%

15. What is your attitude to the idea of legalizing **compulsory **(obligatory) **testing **of school and university students in order to detect and prevent drug addiction?	47.2% ***	39.0%	13.8%	19.6%	69.5%	10.9%

16. In your opinion, should or should not **placebo-controlled clinical studies of medical treatments **be banned in cases where patients' conditions are acute?	28.5%	37.4%	34.1%	23.9%	45.7%	30.4%

17. Do you think that the principle of **informed consent **should have exceptions (for example, in psychiatry or drug addiction treatment)?	54.5%**	34.6%	10.9%	30.4%	56.5%	10.9%

18. Do you think that it is morally permissible, or impermissible, to share **information about those who are mentally ill or are addicted to drugs **with law enforcement services, at their request, in order to support safety in the community?	55.7%**	35.4%	8.9%	24.0%	63.0%	13.0%

19. Do you think that drug addiction is an illness requiring medical treatment (like other illnesses), or is drug addiction a deviant behavior that requires rehabilitation?	75.6%	16.3%	8.1%	73.9%	8.7%	17.4%

Designations: * - p < 0.05; ** - p < 0.01; *** - p < 0.001

Survey findings indicate that attitudes toward the majority of bioethical questions (12 of 19) among Russian and foreign experts differ significantly. Despite the fact that respondents were in agreement in seeing "drug addiction [as] an illness requiring medical treatment (like other illnesses)," instead of "a deviant behavior that requires rehabilitation," which would seem to suggest that they would also share attitudes toward the importance of observing principles of bioethics, this is not reflected in practice.

Significantly different attitudes on the part of Russian and foreign drug addiction treatment professionals toward bioethical problems confronted in narcology were identified in regard to: the need to observe confidentiality; informed consent; the permissibility of compulsory treatment; and substitution therapy. More than half (55.7%) of the Russian specialists surveyed as compared to 24% of foreign specialists (p < 0.01) felt that information about mentally ill patients and drug addicts could be given to law enforcement services "in order to support safety in the community." This suggests that one of the tendencies in Russian narcology is to permit infringement of the principle of confidentiality despite voicing support for it. According to a sociological study [19] that surveyed drug users, infringement of confidentiality in Russian narcology is more the rule than the exception." One in three (34%) faced disclosure of a confidential diagnosis. Respondents believed that information about a diagnosis was most likely to be obtained by the police (52.5%) and relatives (49.8%), or, more rarely, by an educational institution (5.0%) and place of employment (4.5%). Last year, in various regions of the country, patients were systematically deprived of their driver's licences solely due to the fact that they were registered with a drug treatment clinic. In violation of bioethical principles, patient information had been handed over to agencies of law enforcement.

Similar "bioethical nihilism" was seen in respondent attitudes toward the necessity of observing the principle of "informed consent," a topic that is still being debated. Real differences (p < 0.01) were seen between Russian and foreign addiction treatment professionals on this point. Among Russian specialists, 54.5% (in comparison with 30.4% from other countries) felt that exceptions can be made to the principle of informed consent in psychiatry or drug addiction treatment."

The problem of putting the principle of "informed consent" into practice has to do with the fact that in the Russian Federation patients with alcohol or drug dependency, in the course of receiving medical narcological care, either are not given all the information necessary for responsible decision-making about the choice of therapy, or receive it in the distorted form. This has to do first and foremost with the practice of so-called "coding," where the informed consent is constructed in such a way that the doctor misleads the patient about the essence (mechanisms) of the technique [4,7,11]. The patient is informed that "a substance that blocks opioid receptors will be administered" or "brain activity related to the longing for psychoactive substances" will be altered or there will be "coding for a dose" or "the subconscious image of illness" will be destroyed. Informed consent in such cases consists in the patient signing a paper confirming that if he willfully violates the regimen by using drugs or alcohol, he risks seriously damage to his health and even death. For ethical reasons and due to its unscientific nature, this technique is prohibited within the international narcological community.

The question of compulsory treatment in narcology also falls within the purview of bioethics and is a focus of attention within the scientific community, which views it in terms of one of the fundamental principles of modern bioethics - the autonomy principle [10,21-23]. World Health Organization recommendations devoted to treating drug addition [24] emphasize that "In line with the principle of autonomy, patients should be free to choose whether to participate in treatment." The guidelines go on to state that "In situations where opioid-dependent individuals are convicted of crimes related to their opioid use, they may be offered treatment for their opioid dependence as an alternative to a penal sanction," however, "Such treatment would not be considered compulsory."

Survey results showed significant differences (p < 0.001) between attitudes toward compulsory treatment of drug addicts held by Russian and foreign narcology professionals, with 62.6% and 28.3% respectively supporting this practice.

The problem of opioid substitution therapy (OST) [11,16,25-27], which is covered by WHO treatment standards, has emerged in recent years as a new question for bioethics and medical law. According to opponents of OST [3,25], a number of cardinal ethical problems place it beyond the bounds of morality. This has primarily to do with the idea that it is unethical to "treat an illness (drug addiction), knowing that it will continue." Secondly, there is the question of the ethical permissibility of "offering someone one drug to help him stop taking others so that he will became less dangerous to those around him." Thirdly, there is the fact that the ideology of harm reduction programs" (including, OST) espouses a "'more respectful' attitude toward addicts than any medical approach." Supporters of OST argue that this approach promotes such humane goals as improving the patient's "quality of life" and reduces the risk osf overdose, suicidal behavior, mortality, criminal and risky behavior, and so forth. Surely such considerations are not irrelevant to biomedical ethics. Indeed, it could be argued that depriving patients of access to OST is a violation of bioethical principles.

The study showed Russian and foreign experts in drug addiction treatment differing considerably when it came to OST as well (p < 0,001). The overwhelming majority of non-Russian experts (93.5%) support OST as opposed to barely half (51.2%) of Russians working in the field.

It can thus be presumed that the medical community's particular attitudes toward the problems of narcology described above are conditioned by a sense of civic duty that has taken shape along with the suppression of biomedical ethics and concepts of humanism, justice and the wellbeing of the patient.

It is shocking to compare respondent answers to the question about forbidding mentally ill women from having children and the possibility of forcing them to submit to sterilization. Among Russian experts, 40.6% felt these practices were permissible compared to 10.9% of foreign experts (p < 0.001). It is worth noting that this aspect of medical ethics has long since been unequivocally resolved, although this is not reflected in the responses by the Russian narcology professionals.

In addition to the attitudinal differences relating to bioethical problems specific to narcology, the study revealed differences in other areas of bioethics as well. For example, results indicate substantial differences (p < 0.01) in attitudes toward euthanasia - one of the controversial questions facing contemporary bioethics [28-31]. Foreign experts were one-and-a-half times more likely to support euthanasia (69.6% versus 41.5%). It is also interesting to correlate attitudes toward euthanasia with attitudes toward specifically narcology-related bioethical problems. In the Russian sample, attitudes toward euthanasia had the strongest correlation with attitudes toward OST, a connection not seen in the sample of foreign experts, whose responses on this subject strongly negatively correlated with attitudes toward the principles of confidentiality and "informed consent."

Significant differences between the samples were also seen in attitudes toward the permissibility of terminating pregnancy through abortions (p < 0.01), with 95.7% of foreign professionals supporting permissibility versus 77.7% of Russians, and toward compulsory drug testing of students and schoolchildren (p < 0.001), with 47.2% of Russian and 19.6% of foreign respondents expressing support. There were also differences among respondents (p < 0.05) in attitudes toward preventing the transmission of HIV among drug users by providing access to needle and syringe exchange (87% of foreigners favored this practice versus 73.5% of Russian respondents), toward the need to expand access to narcotic analgesics to cancer patients with pain syndrome (97.8% of foreign respondents were in favor versus 87% of Russian ones), and toward sex change operations (76.1% of foreign respondents were in favor versus 61% of Russian ones).

## Conclusions

This survey of bioethical attitudes among Russian and foreign drug addiction treatment professionals points to significant differences both in regard to bioethical questions specific to narcology and most general questions confronting bioethics. It can be assumed that the cardinal differences between the models for providing narcological care in the Russian Federation and in the majority of other countries of the world (based on the WHO principle) is largely a function of differences in the bioethical attitudes among narcology professionals revealed in this study.

Russian narcology developed as a part of what was known as "punitive Soviet psychiatry," within which psychiatric practice was used for political purposes and patient rights were frequently violated. Furthermore, a large number of doctors specializing in the field of narcology had no training in psychiatry. The 1992 law, "Concerning Psychiatric Care and Guaranteeing Patient Rights," enabled the introduction of positive and fundamental change within psychiatry - patient rights were guaranteed and adherence to ethical norms became standard practice. This law, however, was not applied to the practice of narcology. Those afflicted with drug addiction and alcoholism continue to be deprived of the right to humane treatment, legal protection, and confidentiality. Doctors working in narcology in isolation from the psychiatric community continue to favor coercive measures toward patients. This appears to be why their bioethical views differ so much from those of their international colleagues.

## Additional Information

A Russian translation of this article has been provided by author Vladimir D. Mendelevich [see Additional file 1].

## Competing interests

The author declares that they have no competing interests.

## Supplementary Material

Additional file 1 **Russian translation of 'Bioethical differences between drug addiction treatment professionals inside and outside the Russian Federation'**. Click here for file
