# Supplementary material for: Bioethical differences between drug addiction treatment professionals inside and outside the Russian Federation
Source: Harm Reduct J. 2011 Jun 10;8:15. doi: 10.1186/1477-7517-8-15 (PMC3123191; doi:10.1186/1477-7517-8-15)
Supplement: Additional file 1 — Russian translation of 'Bioethical differences between drug addiction treatment professionals inside and outside the Russian Federation'. [file 1477-7517-8-15-S1.PDF]

АЛЬТЕРНАТИВНОСТЬ БИОЭТИЧЕСКИХ ПРЕДПОЧТЕНИЙ РОССИЙСКИХ И ИНОСТРАННЫХ НАРКОЛОГОВ

Владимир Давыдович Менделевич

Казанский государственный медицинский университет,

кафедра медицинской и общей психологии.

420012, г.Казань, ул.Бутлерова, 49, e-mail: mend@tbit.ru

Резюме. В статье приводятся результаты социологического исследования 338 специалистов, оказывающих наркологическую помощь. Сравниваются биоэтические предпочтения российских и иностранных специалистов из 18 стран мира. Делается вывод о том, что биоэтические приоритеты отечественных и зарубежных специалистов достоверно различаются. Это касается несходства отношения к необходимости соблюдения принципов конфиденциальности, информированного согласия, принудительному лечению, терапии агонистами опиоидов, обязательному тестированию учащихся на предмет употребления психоактивных веществ, запрету психическим больным иметь детей, программам снижения вреда (процедуре обмена игл и шприцев), эвтаназии, абортам. Делается вывод о том, что кардинальное несходство моделей оказания наркологической помощи в Российской Федерации и в большинстве стран мира обусловлено выявленными альтернативными биоэтическими предпочтениями наркологов.

Ключевые слова: наркология, биоэтика.

Российская и мировая наркология со второй половины XX века начали развиваться в разных направлениях. Фактически отечественная наркология перестала быть частью мировой. Их сегодня кардинально отличают научные взгляды на природу зависимостей, их нейробиологические или психопатологические основы, на стандарты терапии и методы организации наркологической помощи [2, 7, 9, 12, 16-19, 23, 28, 31, 35, 37, 39]. Известно, что в 1976 году отечественная наркология разорвала «пуповину», связывавшую ее с психиатрией, и выделилась в самостоятельную дисциплину. Из квалификационных требований к специалистам было изъято условие обязательности базового психиатрического образования. В отличие от этого, в подавляющем большинстве стран мира наркология эволюционировала в рамках психиатрии. Можно предполагать, что основной причиной кардинальных различий российской и мировой наркологии на современном этапе являлся факт отдаления советской наркологии от психиатрии, создание условий для репрессивных мер по отношению к больным.

Параллельно с вышеперечисленными тенденциями в отечественной наркологии происходила постепенная ревизия биоэтических и деонтологических норм [11, 13, 36]. При декларировании необходимости соблюдения принципов медицинской этики применявшиеся на практике процедуры противоречили биоэтическим требованиям. Фактически наркологически больной даже в сравнении с психически больным не мог пользоваться всеми правами пациента. Он подвергался дискриминации и стигматизации как в обществе, так и в условиях медицинских наркологических учреждений [10, 14, 16, 21]. Медиками он нередко воспринимался как девиантная, или криминальная личность, вследствие чего в советский период предлагалось применять по отношению к нему меры принуждения, изоляции и перевоспитания.

Современная отечественная наркология, относится к одной из немногочисленных медицинских специальностей, в которой этико-деонтологические приоритеты врачей и иных оказывающих помощь

профессионалов продолжают существенно влиять на процессы диагностики, терапии и реабилитации [17]. Несмотря на то, что наркология фактически признается составной частью психиатрии, на нее не всегда однозначно распространяются нормы биоэтики и медицинского права, используемые в психиатрии [13]. До сих пор ведутся дискуссии о правомерности и целесообразности экстраполяции норм, диктуемых Законом РФ «О психиатрической помощи и гарантиях прав граждан при ее оказании» [6] на наркологически больных [22].

Возможно, данный факт можно интерпретировать как результат принципиально различного отношения врачей к психическим и наркологическим заболеваниям и, как следствие, к соблюдению общепринятых биоэтических принципов. По данным социологических исследований [12], большое число (почти половина) отечественных врачей психиатров-наркологов однозначно не относят наркоманию и алкоголизм к психическим расстройствам или заболеваниям, видя ее причину в «распущенности» человека. Видимо именно поэтому 54,5% наркологов называют «наиболее эффективными методами лечения наркомании» религию.

Гипотезой настоящего исследования явилось предположение о том, что имеются альтернативные особенности структуры биоэтических и этико-деонтологических приоритетов у российских и зарубежных специалистов, участвующих в оказании наркологической помощи. В связи с этим была поставлена и сформулирована **цель** - с помощью социологического подхода и с использованием соответствующего инструментария изучить специфику отношения отечественных и зарубежных специалистов, участвующих в оказании наркологической помощи, к различным актуальным проблемам современной биоэтики и медицинского права. Были выбраны общие для медицинских специальностей спорные биоэтические вопросы, а также специфические для психиатрии и наркологии. В число первых вошли проблемы отношения к эвтаназии, абортам, хирургической смене пола, клонированию, получению плацентарных стволовых клеток, трансплантации органов, плацебо-

контролируемым клиническим испытаниям, ответственности ВИЧ-инфицированного за появление патологии, программам снижения вреда (обмену игл и шприцев, рекомендациям по использованию кондомов). Ко вторым были причислены вопросы, связанные с отношением респондентов к принудительному лечению в наркологии, обязательному (недобровольному) тестированию студентов и школьников на предмет употребления психоактивных веществ, опиоидной заместительной терапии, допустимости разглашения врачебной тайны (конфиденциальности) и нарушений принципа информированного согласия в психиатрии и наркологии, допустимости запрета для душевнобольных на деторождение.

Для проведения социологического обследования была создана анкета, состоявшая из 19 вопросов (русскоязычный и англоязычный варианты). Исследование проводилось анонимно. Метод изучения безвыборочный. В качестве респондентов выступили специалисты, участвующие в оказании наркологической помощи (врачи-психиатры, наркологи и др.), пожелавшие принять участие в данном исследовании. Анкета была разослана по электронной почте, ответы также принимались по электронной почте. Анкеты рассылались на сайты наркологических диспансеров и центров различных регионов России, кафедр психиатрии, наркологии, психотерапии ВУЗов страны, а также на сайты аддиктологических, психиатрических и медицинских Ассоциаций, кафедр психиатрии Университетов и Центров по лечению зависимостей, расположенных в разных странах мира. Всего было разослано более 1000 анкет (700 по РФ и около 300 по странам мира). Заполненные 264 русскоязычные и 92 ангоязычные анкеты были направлены респондентами в адрес исследователей. Были получены заполненные анкеты из 18 стран мира: Австралии, Бельгии, Бразилии, Великобритании, Вьетнама, Германии, Израиля, Италии, Канады, Китая, Латвии, Македонии, Нидерландов, США, Таиланда, Франции, Хорватии, Черногории. Корректно оказались заполненными и вследствие этого подверглись статистической обработке 246 русскоязычных и 92 ангоязычные анкеты. Таким образом, выборка составила 338 человек. В

исследовании приняло участие 138 мужчин (40,8%) и 200 женщин (59,2%). Стаж работы по профилю колебался от 1 до 25 лет.

Как показали **результаты исследования**, позиции респондентов по отношению к различным дискутабельным вопросам биоэтики и медицинского права распределились следующим образом (таблица 1).

Таблица 1.

Отношение респондентов к различным биоэтическим проблемам

	**Российские (n = 246)**			**Иностранные (n = 92)**		
	за	против	затр. с ответом	за	против	затр. с ответом
1. Отношение к узакониванию эвтаназии	41,5%**	38,6%	19,9%	69,6%	13,0%	17,4%
2. Поддержка процедуры хирургической смены пола человеку, у которого диагностирован транссексуализм и у которого не обнаружено психических расстройств	61,0%*	27,6%	11,4%	76,1%	13,0%	10,9%
3. Отношение к гомосексуализму как к болезни, требующей лечения	21,1%	62,2%	16,7%	13,0%	76,1%	10,9%
4. Поддержка разрешения на проведение экспериментов по клонированию человека	27,6%	44,3%	28,1%	32,6%	37,0%	30,4%
5. Допустимость прерывания беременности (аборта) на ранних этапах по запросу женщины, у которой нет медицинских показаний к прерыванию беременности	77,7%**	15,0%	7,3%	95,7%	4,3%	0%
6. Допустимость процедуры получения плацентарных стволовых клеток	70,3%*	12,6%	17,1%	87,0%	4,3%	8,7%
7. Отношение к необходимости расширения доступа онкологически больных с болевых синдромом к использованию наркотических анальгетиков	87,0%*	8,5%	4,5%	97,8%	2,2%	0%
8. Отношение к ВИЧ-инфицированному пациенту как к повинному в появлении собственного заболевания	19,5%	43,1%	37,4%	10,9%	47,8%	41,3%
9. Отношение к процедуре трансплантации органов	86,2%	6,1%	7,7%	93,5%	4,3%	2,2%
10. Допустимость внедрения принудительного лечения больных алкоголизмом и наркоманией по медицинским показаниям	62,6%***	29,3%	8,1%	28,3%	71,7%	0%
11. Отношение к необходимости запрета психически больным женщинам рожать детей и возможности проведения стерилизации	40,6%***	37,8%	21,6%	10,9%	78,3%	8,6%
12. Отношение к идее внедрения заместительной терапии	51,2%***	31,3%	17,5%	93,5%	4,3%	2,2%

наркомании, предполагающей назначение больным наркоманией по медицинским показаниям наркосодержащих лекарств						
13. Допустимость профилактики полового пути передачи ВИЧ среди подростков с помощью пропагандирования использования «защищенного секса» с применением презервативов (кондомов)	89,8%	4,5%	5,7%	93,5%	2,2%	4,3%
14. Допустимость профилактики инъекционного пути передачи ВИЧ среди наркозависимых с помощью расширения доступа наркопотребителей к обмену использованных игл и шприцев на новые	73,5%*	16,7%	9,8%	87,0%	8,7%	4,3%
15. Отношение к идее узаконивания принудительного (обязательного) тестирования студентов и школьников на предмет выявления и предупреждения наркомании	47,2%***	39,0%	13,8%	19,6%	69,5%	10,9%
16. Отношение к наложению запрета на проведение плацебо-контролируемых клинических испытаний лекарственных средств в случае изучения острых состояний у пациентов	28,5%	37,4%	34,1%	23,9%	45,7%	30,4%
17. Отношение к тому, что принцип информированного согласия должен иметь исключения в психиатрии и наркологии	54,5%**	34,6%	10,9%	30,4%	56,5%	10,9%
18. Допустимость того, чтобы сведения о психически и наркологически больных передавались в правоохранительные органы в целях поддержания безопасности в обществе	55,7%**	35,4%	8,9%	24,0%	63,0%	13,0%
19. Отношение к наркомании как к болезни, требующей медикаментозного лечения, а не как к девиантному поведению, требующему перевоспитания	75,6%	16,3%	8,1%	73,9%	8,7%	17,4%

Обозначения: * - $p<0{,}05$; ** - $p<0{,}01$; *** - $p<0{,}001$

Как следует из полученных данных социологического исследования, по отношениею к большинству биоэтических проблем (12 из 19) ответы российских и зарубежных специалистов различались достоверно. Несмотря на тот факт, что респонденты сходились во мнении, что «наркомания является заболеванием, требующим медикаментозного лечения, а не перевоспитания пациента» и можно было ожидать идентично единого отношения к

необходимости соблюдения принципов биоэтики, на деле результаты оказались различными.

Было выявлено достоверно различное отношение российских и зарубежных наркологов к таким биоэтическим проблемам наркологии как: необходимости соблюдения конфиденциальности, информированного согласия, допустимости принудительного лечения и опиоидной заместительной терапии. Более половины (55,7%) опрошенных отечественных специалистов по сравнению с 24% иностранных ($p<0,01$) допускали, что сведения о психически и наркологически больных могут передаваться в правоохранительные органы «в целях поддержания безопасности в обществе». То есть позволительно признать, что одной из тенденций российской наркологии является поощрение нарушения принципа конфиденциальности при декларировании его незыблемости. По данным социологического исследования потребителей инъекционных наркотиков [14] нарушение конфиденциальности в отечественной наркологии является «скорее правилом, чем исключением». Каждый третий (34%) сталкивался с разглашением тайны своего диагноза. По мнение опрошенных чаще всего информация о диагнозе оказывалась у сотрудников милиции (52,5%) и родственников (49,8%), реже – по месту учебы (5,0%) и работы (4,5%). В течение последнего года в различных регионах страны состоящие на учете больные в массовом порядке были лишены прав на вождение автомобиля только на основании факта нахождения на диспансерном учете. Сведения о больных были переданы в правоохранительные органы с нарушением этико-деонтологических принципов.

Сходный «биоэтический нигилизм» был зарегистрирован при оценке отношения респондентов к необходимости соблюдения принципа «информированного согласия», вокруг которого до настоящего времени ведется дискуссия [1, 13]. Были обнаружены достоверные различия ($p<0,01$) между позицией по данному вопросу российских и зарубежных наркологов. 54,5% отечественных специалистов (в сравнении с 30,4% иностранных) высказались за то, что «принцип информированного согласия должен иметь исключения в

психиатрии и наркологии».

Проблема реализации принципа «информированного согласия» в РФ заключается в том, что больной с алкогольной или наркотической зависимостью в процессе оказания ему медицинской наркологической помощи либо не получает всего объема информации, необходимого ему для осознанного и ответственного принятия решения о выборе терапии, либо получает ее в искаженном виде. В первую очередь это относится к практике т.н. «кодирования», при котором информированное согласие строится на предоставлении пациенту врачом ложных данным о сути (механизмах) действия этой методики [9, 16]. Больного ставят в известность о том, что ему будет «введено вещество, блокирующее опиоидные рецепторы» или «будет изменена деятельность его головного мозга, которая снимет тягу к психоактивному веществу» или произойдет «кодирование на дозу» или «разрушится подсознательный образ болезни». Реализация информированного согласия в подобных случаях заключается в том, что пациент подписывает бумагу, в которой подтверждает согласие, что в случае добровольного нарушения им режима и принятия дозы спиртного (наркотика) его здоровье может подвергнуться серьезному риску ухудшения вплоть до летального исхода. По этическим соображениям и вследствие ненаучности данная методика запрещена мировым наркологическим сообществом.

Проблема принудительного лечения в наркологии также входит в круг биоэтических, находится в центре внимания научной общественности и рассматривается сквозь призму соблюдения одного из основополагающих принципов современной биоэтики — принципа автономности [8, 15, 27, 29, 41-43]. В рекомендациях Всемирной организации здравоохранения, посвященных терапии наркомании [43] прямо указывается на то, что «в соответствии с принципом автономности, у пациента должна быть свобода выбора — участвовать или не участвовать в лечении». В ситуациях же, «когда лица осуждены за преступления, связанные с употреблением наркотиков, им может быть предложено лечение в качестве альтернативы уголовному наказанию»,

однако «такое лечение не считается принудительным».

Результаты социологического исследования показали достоверные различия ($p<0,001$) между отношением к допустимости принудительного лечения наркологически больных российских и зарубежных наркологов. Соответственно 62,6% и 28,3% поддержали данную процедуру.

Новой проблемой биоэтики и медицинского права стала в последние годы проблема опиоидной заместительной терапии (ОЗТ) [5, 12, 13, 35, 37, 38], входящей в ВОЗовские стандарты лечения. ОЗТ – это назначение больным с опиоидной зависимостью (героиновой наркоманией) в медицинских учреждениях под врачебным контролем обоснованных их наркологическим состоянием строго определенных доз препаратов, являющихся агонистами опиоидов (аналогами наркотических веществ из той же фармакологической группы) [42, 43].

По мнению противников ОЗТ [5], существует ряд кардинальных этических проблем, ставящих ее вне рамок морали. Во-первых, это относится к проблеме этической оценки «отказа от лечения болезни (наркомании), понимая, что она продолжается». Во-вторых, к этической допустимости «предлагать человеку один наркотик для того, чтобы он отказался от других и стал менее опасен для окружающих». В-третьих, к тому, что «идеология программ «снижения вреда» (в том числе, ОЗТ) заявляет «более уважительное» отношение к наркопотребителям/наркоманам, чем любой медицинский подход». Сторонники ОЗТ аргументируют свою позицию тем, что в рамках подобной терапии ставятся такие гуманные цели как улучшить «качество жизни» пациента, снизить риск передозировок, суицидального поведения и летальных исходов, криминального и рискованного поведения и пр. Они не могут рассматриваться как выходящие за рамки биомедицинской этики. И, наоборот, отсутствие доступа пациента к ОЗТ рассматривается как нарушение принципов этики и деонтологии.

По результатам социологического исследования мнения по поводу ОЗТ российских и зарубежных наркологов достоверно различались ($p<0,001$). За

ОЗТ выступило подавляющее большинство (93,5%) специалистов из разных стран мира и чуть больше половины (51,2%) отечественных наркологов.

Таким образом, можно предполагать, что особое отношение медицинского сообщества к вышеперечисленным проблемам наркологии обусловлено сформировавшейся стратегией гражданственности и подавления стратегии биомедицинского этики с понятиями гуманизма, справедливости и блага для больного.

Шокирующими оказались результаты сравнения ответов респондентов на вопрос о допустимости запрета психически больным женщинам рожать детей и возможности проведения стерилизации. За допустимость данной процедуру высказались 40,6% российских специалистов и 10,9% иностранных (достоверность различий — $p<0,001$). Следует признать, что этот аспект медицинской этики давно считается однозначно разрешенным [3, 4, 6, 20, 24-26, 30, 32-34, 40], что не следует из приведенных данных социологического исследования российских наркологов.

Помимо обнаруженных различий в позициях специалистов из России и стран мира по специфически наркологическим биоэтическим проблемам, по результатам социологического исследования были зарегистрированы и иные кардинальные несходства позиций. Так, было выявлено, что отношение к эвтаназии - одной из наиболее острых этико-деонтологических проблем современной биоэтики — представителей двух обследованных групп достоверно различалось ($p<0,01$). Среди иностранных специалистов сторонников эвтаназии оказалось в полтора раза больше (69,6% и 41,5% соответственно). Интерес представлял корреляционный анализ взаимосвязи отношения к необходимости узаконивания эвтаназии с отношением к специфически наркологическим этико-деонтологическим проблемам. В российской выборке отношение к эвтаназии имело наиболее сильные прямые связи с отношением к ОЗТ, чего не наблюдалось в сравниваемой выборке иностранных специалистов, в которой были обнаружены сильные отрицательные корреляционные связи с отношением к допустимости

нарушения принципов конфиденциальности и «информированного согласия».

Достоверные различия между выборками были зарегистрированы также по отношению к допустимости прерывания беременности - абортам ($p<0,01$), за которое высказались 95,7% иностранных специалистов и 77,7% отечественных; к идее узаконивания принудительного тестирования студентов и школьников на предмет употребления ПАВ ($p<0,001$), за которое выступило 47,2% российских и 19,6% зарубежных респондентов. Были обнаружены достоверные различия выборок ($p<0,05$) по отношению к допустимости профилактики инъекционной передачи ВИЧ среди потребителей наркотиков с помощью доступа к обмену игл и шприцев («за» это оказалось 87% иностранных и 73,5% российских респондентов), к необходимости расширения доступа онкологически больных с болевым синдромом к использованию наркотических анальгетиков («за» это оказалось 97,8% иностранных и 87% российских респондентов), к процедуре хирургической смены пола («за» это оказалось 76,1% иностранных и 61% российских респондентов).

Таким образом, проведенное социологическое исследование биоэтических предпочтений отечественных и зарубежных специалистов, оказывающих наркологическую помощь, позволяет отметить тот факт, что обнаруживаются достоверные различия как по большинству специфических для наркологии, так и по большинству общемедицинских этико-деонтологических проблем. Можно предполагать, что кардинальное несходство моделей оказания наркологической помощи в Российской Федерации и в большинстве стран мира (базирующейся на ВОЗовских принципах) во многом обусловлено выявленными альтернативными биоэтическими предпочтениями наркологов.

ЛИТЕРАТУРА:

1. Агибалова Т.В., Голощапов И.В., Рычкова О.В. Согласие на лечение в наркологии: старая проблема и новый подход. //Наркология. - 2008. -№1.
2. Айзберг О.Р. Этическая обоснованность научных исследований в области наркологии – идеал, процедуры и реальность. http://www.narcom.ru/publ/info/264

3. Биоэтика: вопросы и ответы /под ред.Б.Г.Юдина и П.Д.Тищенко. – М.; 2005. – 64 с.
4. Евтушенко В., Иванюшкин А., Покуленко Т., Тихоненко В. Основы профессиональной этики в психиатрии: принципы, нормы, механизмы /http://www.narcom.ru/cabinet/online/92.html
5. Елшанский С.П. Некоторые этические и психологические проблемы реализации программ «снижения вреда» среди потребителей наркотиков. //Вопросы наркологии. – 2003. - №2. – с.36-51.
6. Закон о психиатрической помощи и гарантиях прав граждан при ее оказании /Постатейный комментарий к Закону РФ. Под редакцией В.П.Котова. – М.: «Республика», 1993. – 238 с.
7. Иванец Н.Н. Наркология – предмет и задачи. Современная концепция терапии наркологических заболеваний./Лекции по наркологии. – М.: «Нолидж», 2000. – с.7-15, 134-148.
8. Клименко Т.В. Этические и организационные аспекты недобровольного лечения лиц с наркологическими заболеваниями с учетом национального и зарубежного опыта. //Наркология. - 2007. - №7.
9. Крупицкий Е.М. Краткосрочное психотерапевтическое вмешательство в наркологии с позиции доказательной медицины. //Неврологический вестник. - 2010. - №3. - с.25-27.
10. Левинсон Л., Торбан М. Наркоучет: по закону или по инструкции?: регулирование регистрации потребителей наркотиков в Российской Федерации. - М.: Анахарсис, Библиотека ПравЛит, 2009. - 100 с.
11. Менделевич В.Д. Общественное мнение о психиатрии (правовые и этические аспекты в социологическом ракурсе). //Независимый психиатрический журнал. - 1997. - №1. - с.46-50.
12. Менделевич В.Д. Наркомания и наркология в России в зеркале общественного мнения и профессионального анализа. Казань: Медицина, 2006. - 262 с.
13. Менделевич В.Д. Этика современной наркологии. Казань: «Медицина», 2010. - 218 с.
14. Олейник С. Наркологическая служба глазами потребителей наркотиков. Пенза, 2007. - 33 с.
15. Пелипас В.Е. Этические аспекты наркологии. /Наркология: национальное руководство. М.: «ГЭОТАР-Медиа», 2008. - с.710-715.
16. Райхель Е. Применение плацебо в постсоветском периоде: гносеология и значение для лечения алкоголизма в России. //Неврологический вестник. - 2010. - №3. - с.9-24.
17. Руководство по аддиктологии /под ред. проф.В.Д.Менделевича. СПб.: Речь, 2007. - 768 с.
18. Сиволап Ю.П., Савченков В.А. Злоупотребление опиоидами и опиоидная зависимость - М.: Медицина, 2005. – 301 с.
19. Софронов А.Г. Актуальные проблемы развития отечественной наркологии //Наркология. - 2003. - №3.
20. Тихоненко В.А., Иванюшкин А.Я., Шишков С.Н. Психиатрия, этика и

право. /Руководство по социальной психиатрии. – М.: Медицина, 2001. – с.51-73.
21. Торбан М.Н., Илюк Р.Д., Крупицкий Е.М., Хаймер Р. Исследование представлений врачей-наркологов РФ о работе наркологической службы - СПб.: Изд. СПб НИПНИ им. В.М. Бехтерева, 2010. - 59 с.
22. Цымбал Е.И. Правовое регулирование оказания наркологической помощи: проблемы и пути их решения. Ч.2. //Наркология. – 2005. - №6. – с.8-15.
23. Энтин Г.М., Копоров С.Г. В современной российской наркологии нет парадоксальных принципов, а методики лечения наркологических больных отвечают социально-экономической ситуации в стране и по эффективности не уступают зарубежным //Наркология. - 2005. - №6. - с.61-68.
24. Юдин Б.Г. Социальная справедливость как проблема биоэтики. //http://www.zpu-journal.ru/gumtech/projection/articles/2007/Yudin/
25. Baruch A. Brody N. The Ethics of Biomedical Research: An International Perspective, 1st ed.: New York, Oxford University Press, 1998.
26. Beauchamp T.L., Childress J.F. Principles of Biomedical Ethics. 3-d ed., N.Y., Oxford: Oxford univ. Press, 1989.
27. Caplan A.L. Ethical issues surrounding forced, mandated, or coerced treatment. //Journal of Substance Abuse Treatment. - 2006. - v.31(2). - p.117–120.
28. Carter A., Capps B., Hall W. Addiction neurobiology: Ethical and social implications. Lisbon, European Monitoring Centre for Drugs and Drug Addiction, 2009.
29. Foddy B., Savulescu J. Addiction and autonomy: Can addicted people consent to the prescription of their drug of addiction? //Bioethics. - 2006. - v.20(1). - p.1-15.
30. Francoer R.T. Biomedical Ethics: A guide to decision making — N.Y., etc.: Wiley, 1983.
31. Geppert C.M.A., Bogenschutz M.P. Ethics in substance use disorder treatment //Psychiatric Clinics of North America. - 2009. - v.32(2). - p.283.
32. Geppet C., Roberts L.W. The Book of Ethics: Expert Guidance for Professionals Who Treat Addiction, 2008. - 150 p.
33. Hall W. Ethical eye: drug addiction. //Addiction. – 2006. – v.101 (3). – p.460-461.
34. Kleinig J. Ethical issues in substance use intervention. //Substance Use & Misuse. – 2004. – v.39(3). - p.369-398.
35. Kreek M.J. The addict as a patient. / In Lowinson J.H., Ruiz P., Millman R.B. & Langrod J.G. (Eds.), Substance abuse: A comprehensive textbook (pp. 997 – 1009). Baltimore: Williams & Wilkins. 1992.
36. Mendelevich V. Bioethical preferences of supporters and opponents of agonist opioid therapy in Russia. //Heroin Addiction and Related Clinical Problems 2010; 12(3):33-38.
37. Mendelevich V. Substitution therapy: A New Problem of Biomedical Ethics and Medical Law. //Heroin addiction and related clinical problems. - 2009. -

№11(2). - p.41-46.
38. Pompidou Group. Platform on Ethical issues and professional standards. http://www.coe.int/t/dg3/pompidou/Activities/ethics_en.asp
39. Schipper I., Weyzig F. Ethics for Drug Testing in Low and Middle In Countries: Considerations from European Market, 2008. http://somo.nl/html/paginas/pdf/Ethics_for_Drug_Testing_feb08_NL.pdf
40. Taleff M.J. Advanced Ethics for addiction professionals. - NY: Springer Publishing Company, 2010. http://www.scribd.com/doc/25610881/Advanced-Ethics-for-Addiction-Professionals.
41. Uchtenhagen A.A. Ethical perspectives in caring for people living with addictions: The European experience //Int Rev Psychiatry. - 2010. - v.22(3). - p.274-280.
42. UNODC, WHO. Principles of drug dependence treatment, Vienna, United Nations Office on Drugs and Crime. - 2008.
43. WHO: guidelines for the psychosocially assisted pharmacological treatment of opioid dependence. Geneva, 2009. - 123 p.
